# Molecular Subtype-Associated Response to Cyclophosphamide–Epirubicin–Cisplatin Regimen in Recurrent or Metastatic Adenoid Cystic Carcinoma: A Retrospective Single-Center Study

**DOI:** 10.3390/cancers18111847

**Published:** 2026-06-04

**Authors:** Wenbo Tang, Jiuli Zhou, Wei Zhao, Fengjuan Lin, Liqiong Xue, Ye Guo

**Affiliations:** Department of Oncology, Shanghai East Hospital, School of Medicine, Tongji University, Shanghai 200123, China; 2405872@tongji.edu.cn (W.T.); 103387@tongji.edu.cn (J.Z.); zhaoweioncology@tongji.edu.cn (W.Z.); 2205708@tongji.edu.cn (F.L.)

**Keywords:** recurrent or metastatic adenoid cystic carcinoma, cyclophosphamide–epirubicin–cisplatin, tyrosine kinase inhibitor, salvage chemotherapy, ACC molecular subtype, PIK3CA mutation, bone metastasis

## Abstract

Background/Objectives: Recurrent or metastatic adenoid cystic carcinoma (R/M ACC) has no standard systemic therapy. VEGFR-targeting tyrosine kinase inhibitors (TKIs) are commonly used first-line, though no international standard exists; cisplatin-based chemotherapy is an alternative. We retrospectively reviewed the cyclophosphamide–epirubicin–cisplatin (CEP) regimen to determine whether prior TKI exposure compromises subsequent chemotherapy efficacy. Methods: We studied 31 patients given CEP for progressive R/M ACC (2018–2023). Tumor response was assessed by RECIST 1.1. Molecular subtype was determined by c-MYC/p63 immunohistochemistry (ACC-I, c-MYC-positive/p63-negative; ACC-II, p63-positive/c-MYC-low or negative). Multivariable models used Firth’s penalized likelihood Cox regression. Next-generation sequencing (NGS) was available in 21 of 31 patients. Results: Thirty-one patients were enrolled (median age 48; 17 [54.8%] with prior TKI). At median follow-up of 22.6 months, the objective response rate (ORR) was 19.4%, disease control rate 71.0%, median progression-free survival (PFS) 5.3 months, and median overall survival (OS) 10.3 months. Prior TKI did not lower efficacy: ORR 17.6% vs. 21.4%, PFS hazard ratio 0.76 (*p* = 0.519). All six partial responses occurred in ACC-I tumors (35.3% vs. 0% in ACC-II, *p* = 0.021). In the NGS subset (5 PIK3CA-mutant), PIK3CA mutation (OS HR 6.19, *p* = 0.024) and bone metastasis (OS HR 5.84, *p* = 0.027) remained associated with shorter OS after adjustment. No treatment-related deaths occurred. Conclusions: CEP is active in R/M ACC, and prior TKI exposure did not appear to reduce efficacy. Higher response rates in ACC-I tumors and the apparent PIK3CA-related survival deficit are exploratory observations that need prospective testing before they can guide treatment.

## Simple Summary

Adenoid cystic carcinoma is a rare cancer of the salivary glands. Once it spreads, no drug has been approved for it, and current options help only modestly. Drugs that target tumor blood vessels work for a while but stop working over time, and there is no clear standard for what to give next. We looked back at 31 such patients treated at our hospital with a three-drug chemotherapy combination. Prior blood-vessel-targeting therapy did not reduce how well chemotherapy worked. Tumor shrinkage was seen only in one of the two molecular subtypes—none in the other. In 5 patients, a gene alteration was linked to shorter survival. These numbers are small. Larger prospective studies are needed before any of this should change how patients are treated.

## 1. Introduction

Adenoid cystic carcinoma (ACC) accounts for approximately 1% of head and neck malignancies and 10% of salivary gland cancers, with a slight female predominance and a median age at diagnosis in the sixth decade [1]. Population-based data from the United States show a declining incidence over the past four decades (from 0.41 to 0.25 per 100,000), yet prevalence has increased nearly tenfold—a paradox that reflects the protracted disease course [2]. A recent systematic review of 17,497 patients reported 5- and 10-year overall survival rates of 73.8% and 48.5%, with distant metastasis—primarily to the lungs, bones, and liver—as the most frequent adverse outcome, occurring at a mean of 35 months after diagnosis [3]. Despite its typically indolent growth, ACC carries a cumulative risk of recurrence and metastasis that rises steadily over decades, making long-term follow-up essential for meaningful assessment of systemic therapies.

No systemic therapy has been approved for recurrent or metastatic (R/M) ACC. Current REFCOR guidelines recommend initiating systemic treatment only for rapidly progressive disease (>20% tumor growth within the preceding 6 months), symptomatic burden, or functional threat; polymetastatic patients with indolent disease may be managed with active surveillance for months or years [4]. When systemic therapy is warranted, VEGFR-targeting TKIs are frequently selected as first-line therapy in clinical practice [5]; however, no international standard exists, and current REFCOR guidelines characterize the choice between TKIs and cytotoxic chemotherapy as dependent on clinical context [4]. Two agents have accumulated the strongest evidence: lenvatinib produced an ORR of 15.6% and a median progression-free survival (PFS) of 17.5 months in a phase II trial [6], while axitinib—in the only randomized controlled trial conducted in R/M ACC—significantly prolonged median PFS compared with observation (10.8 vs. 2.8 months; *p* < 0.001) [7]. A recent meta-analysis of 17 prospective trials encompassing 560 patients treated with VEGFR inhibitors reported a pooled ORR of 6% but a stable disease rate of 82%, confirming that disease stabilization rather than tumor regression is the predominant treatment effect; however, grade ≥ 3 adverse events occurred in 53% of patients and dose reductions were required in 59% [8]. Lenvatinib-associated toxicities—particularly fatigue and xerostomia—led to clinically meaningful deterioration in patient-reported quality of life within six months of treatment initiation, despite preserved swallowing and physical functioning [9].

Cisplatin-based combination chemotherapy remains an option for R/M ACC patients with rapid progression or high symptom burden, though the evidence base is limited to small, nonrandomized studies. A systematic review of 25 chemotherapy trials reported single-agent objective response rates (ORRs) of approximately 12%, while the combination of cyclophosphamide, doxorubicin, and cisplatin (CAP) achieved ORRs of approximately 25% across multiple prospective studies [10]. Of note, REFCOR guidelines recommend cisplatin plus vinorelbine as the preferred chemotherapy regimen (grade B recommendation), with CAP listed as an alternative [4]. Among CAP studies, Licitra et al. reported partial responses in 6 of 22 patients (27%) with advanced salivary gland carcinomas including ACC [11], and Tsukuda et al. observed comparable response rates in patients with recurrent ACC treated with platinum–anthracycline combinations [12]. No prospective trial has directly compared TKIs and cytotoxic chemotherapy in R/M ACC. As TKIs are increasingly adopted as first-line systemic therapy, the efficacy of chemotherapy following prior TKI exposure has not been studied, and whether prior TKI exposure influences chemosensitivity remains an open question.

Cardiac safety represents a shared concern across both treatment modalities: VEGFR-targeting TKIs carry an inherent risk of cardiovascular toxicity, including hypertension, cardiac dysfunction, and arterial thromboembolic events [13]; anthracycline-based chemotherapy, conversely, poses a risk of cumulative dose-dependent cardiomyopathy. Epirubicin, a 4′-epi-isomer of doxorubicin, is associated with a significantly lower incidence of cardiotoxicity at equieffective doses [14] and has demonstrated single-agent activity against ACC, with a 10% response rate and clinical benefit in symptomatic patients in an EORTC phase II trial [15].

The aim of this study was to evaluate the efficacy and safety of the CEP regimen (cyclophosphamide, epirubicin, and cisplatin) in patients with progressive R/M ACC, with a specific focus on whether prior TKI exposure influences CEP efficacy. In addition, with the emergence of ACC molecular subtype classification based on c-MYC and p63 immunohistochemistry [16,17] and the availability of NGS data in a subset of patients, we performed post hoc exploratory analyses to examine the association of ACC molecular subtype and genomic alterations with treatment response and survival. All subtype- and genomic-level findings are therefore hypothesis-generating and intended to inform future prospective evaluation.

## 2. Materials and Methods

### 2.1. Study Design and Patients

This single-center retrospective cohort study enrolled consecutive patients with progressive or symptomatic recurrent/metastatic adenoid cystic carcinoma (R/M ACC) treated with the CEP regimen at Shanghai East Hospital, School of Medicine, Tongji University, between 1 January 2018 and 31 December 2023. Given the rarity of R/M ACC, all eligible patients treated with the CEP regimen were included regardless of line of systemic therapy. Eligibility criteria included age ≥ 18 years and histologically confirmed R/M ACC with documented disease progression or symptomatic disease requiring systemic therapy. The CEP regimen consisted of cyclophosphamide (500 mg/m^2^ on day 1), epirubicin (70 mg/m^2^ on day 1), and cisplatin (25 mg/m^2^ on days 1 and 2), administered every three weeks for up to six cycles. Of 33 consecutive patients with R/M ACC who initiated CEP during this period, 2 were excluded owing to loss to follow-up before the first response assessment, yielding an analytic cohort of 31 patients. The study was conducted in accordance with the Declaration of Helsinki and approved by the Ethics Committee of Shanghai East Hospital (approval number: 2024YS-113; date of approval: 26 June 2024). Given the retrospective nature of the study, the requirement for informed consent was waived by the Ethics Committee.

### 2.2. Data Collection

Patient demographics, disease characteristics, and treatment details were extracted from medical records. Clinical variables included age, sex, primary tumor site, histologic subtype (cribriform, tubular, solid, or mixed), initial disease presentation (de novo metastatic vs. recurrence after initial curative-intent treatment), primary curative-intent treatment for the original disease (surgery, radiotherapy, or both), sites of metastasis (lung, liver, bone, and others), prior lines of systemic therapy, prior exposure to VEGFR-targeting TKIs (anlotinib, apatinib, and lenvatinib), and prior exposure to other systemic therapy classes (cytotoxic chemotherapy, anti-PD-1 monoclonal antibodies, anti-EGFR monoclonal antibodies, and investigational/clinical-trial agents). Histologic subtype was collected from original pathology reports; classification beyond the diagnosis of adenoid cystic carcinoma was not consistently available, and no central histologic re-review was performed. Similarly, because prior TKI therapy was typically administered at outside institutions before referral, detailed TKI treatment parameters—including duration, best response, reason for discontinuation, and washout interval before CEP—were inconsistently documented across the cohort and could not be analyzed systematically. Tumor response was assessed according to the Response Evaluation Criteria in Solid Tumors (RECIST) version 1.1 by the treating investigators. Adverse events were graded per the Common Terminology Criteria for Adverse Events (CTCAE) version 5.0.

### 2.3. Molecular Profiling and ACC Subtype Classification

Molecular subtyping of ACC was based on immunohistochemical (IHC) staining for c-MYC and p63 as reported in the original pathology records of each patient, according to the two-marker classification system established by Ferrarotto et al. [16] and validated by Hanna et al. [17]. Tumors with c-MYC overexpression and absent p63 were classified as ACC-I, whereas tumors with p63 overexpression and low or absent c-MYC were classified as ACC-II. The ACC-I/ACC-II assignments were reconstructed by the investigators from the c-MYC and p63 staining results reported by the referring institutions; Because IHC was performed at the originating laboratories, the antibody clones, staining platforms, and scoring protocols varied across cases and could not be retrieved; predefined positivity thresholds (e.g., percentage of stained cells or H-score cutoffs) were also inconsistently documented and could not be reconstructed retrospectively. No central re-review or formal interobserver agreement analysis was performed at our institution. On the basis of the available c-MYC and p63 results, all 31 tumors could be assigned to one of the two molecular subtypes (17 ACC-I, 14 ACC-II), with no unclassifiable cases. This IHC-based classification has demonstrated strong concordance with transcriptomic profiling and robust prognostic stratification across multiple independent cohorts [17,18].

Next-generation sequencing was performed using the FoundationOne CDx assay (Foundation Medicine, Inc., Cambridge, MA, USA) as part of routine clinical practice. This hybrid-capture-based assay interrogates 324 cancer-related genes; per the manufacturer’s specifications, the assay targets a median coverage depth of >500× and applies variant-calling thresholds of ≥5% allele frequency for substitutions and short indels. Genomic data were available for 21 of the 31 enrolled patients. NGS could not be performed in the remaining 10 patients because adequate archival tumor tissue was not available for sequencing, primarily reflecting the inability to retrieve tissue blocks from outside referral institutions where primary surgery had been performed. Key alterations analyzed included MYB/NFIB fusion, NOTCH1, PIK3CA, TP53, and ARID1A mutations.

### 2.4. Outcomes

The primary outcomes were objective response rate (ORR), progression-free survival (PFS), and overall survival (OS). ORR was defined as the proportion of patients achieving a complete response (CR) or partial response (PR) per RECIST 1.1. PFS was defined as the time from initiation of the CEP regimen to disease progression or death from any cause, whichever occurred first. OS was defined as the time from treatment initiation to death from any cause. Patients who had not experienced the event of interest were censored at the date of last follow-up. Median follow-up was calculated using the reverse Kaplan–Meier method.

### 2.5. Statistical Analysis

Continuous variables were summarized as medians with ranges, and categorical variables as frequencies with percentages. Binomial proportions for response rates (ORR and DCR) were reported with Wilson score 95% confidence intervals. Categorical variables were compared using Fisher’s exact test. Survival outcomes were estimated using the Kaplan–Meier method and compared by the log-rank test. Median follow-up was calculated using the reverse Kaplan–Meier method.

Univariable Cox proportional hazards regression was performed to evaluate candidate prognostic factors for PFS and OS. Covariates for multivariable analysis were pre-specified based on established prognostic significance in ACC and the clinical objectives of this study. Given the limited number of events relative to covariates, Firth’s penalized likelihood Cox regression was used for all multivariable models to reduce finite-sample bias [19]. Events per variable ranged from 4.3 to 12.5 across all multivariable models; given the overall sample size, all multivariable findings are presented as exploratory. To minimize selection bias, univariable Cox regression for genomic variables in the NGS subgroup was pre-specified for the three most frequently altered genes (MYB fusion, NOTCH1, and PIK3CA mutations). To explore the impact of treatment heterogeneity, line of CEP therapy was incorporated as a covariate in a supplementary multivariable Cox model (Supplementary Table S1). Disease-burden indicators (ECOG performance status, liver and bone metastasis) were evaluated within Cox regression rather than as separate subgroup analyses, given the limited cohort size. Recurrence/metastatic TNM (rTNM) staging was not entered as a covariate in survival models because M-status at CEP initiation was near-constant in this cohort (M1 in 29 of 31 patients, 93.5%; M0 in only 2), providing insufficient variance for Cox modelling, and detailed T/N classification at recurrence was not consistently retrievable across the cohort. Missingness was concentrated in two variables: histologic subtype (16/31, 51.6% unspecified) and genomic alterations (10/31, 32.3% without NGS data). For each multivariable model, patients with missing values on any included covariate were excluded (complete-case analysis). Multiple imputation was not performed because the missingness in molecular variables was structural (tumor tissue was not available for sequencing in 10 patients) rather than at random, violating the missing-at-random assumption required for valid imputation. Histologic subtype was therefore not entered as a covariate in any multivariable model. Sensitivity analyses restricted to patients with complete histologic or molecular data were not feasible given the resulting reduction in sample size. All tests were two-sided, with *p* < 0.05 considered statistically significant. Analyses were performed using R version 4.3.1 (R Foundation for Statistical Computing, Vienna, Austria).

## 3. Results

### 3.1. Baseline Characteristics

**Figure 1:**
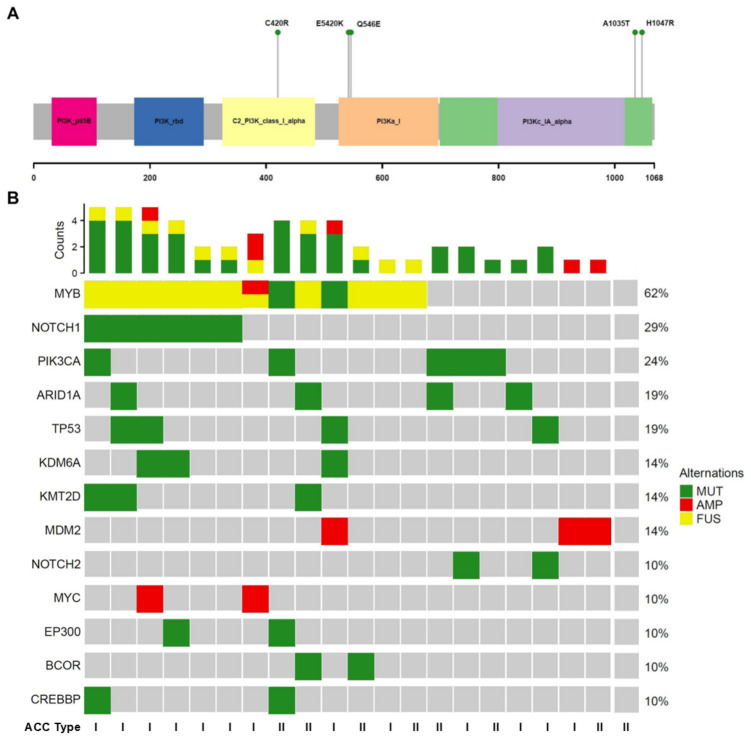
Genetic alterations in the NGS subgroup (*n* = 21). (**A**) Schematic representation of mutation sites within the PIK3CA gene. Lollipop plot showing the distribution and functional domains affected by PIK3CA mutations identified in this cohort. (**B**) Mutational landscape (oncoprint) showing alterations with a frequency of ≥10% as determined by next-generation sequencing (FoundationOne CDx). Each column represents one patient; rows represent individual genes. The ACC molecular subtype (ACC-I vs. ACC-II) is annotated at the bottom. ACC, adenoid cystic carcinoma; AMP, amplification; FUS, fusion; MUT, mutation.

Thirty-one patients were enrolled, with a median age of 48 years (range, 25–76). The cohort was evenly distributed by sex (16 females, 51.6%). Primary tumors arose from minor salivary glands in 17 patients (54.8%), major salivary glands in 12 (38.7%), and lacrimal glands in 2 (6.5%). A solid histologic component was present in 10 patients (32.3%), cribriform or tubular patterns in 5 (16.1%), and histologic subtype was unavailable in 16 patients (51.6%). Lung was the most common metastatic site (20 patients, 64.5%), followed by bone (10, 32.3%) and liver (9, 29.0%). Ten patients (32.3%) received CEP as first-line systemic therapy, while 21 (67.7%) had received prior systemic therapy, including 17 (54.8%) with prior TKI exposure (most commonly anlotinib [*n* = 9] and apatinib [*n* = 8]; Table 1). Based on c-MYC and p63 immunohistochemistry, 17 patients (54.8%) were classified as ACC-I and 14 (45.2%) as ACC-II. NGS data were available for 21 patients; the most frequent genomic alterations were MYB fusion (62%), NOTCH1 mutations (29%), and PIK3CA mutations (24%), additional alterations with a frequency ≥ 10% included TP53, ARID1A, KDM6A, KMT2D, MDM2, NOTCH2, MYC, EP300, BCOR, and CREBBP (Figure 1). Within the NGS subgroup (*n* = 21), 11 patients (52.4%) had prior TKI exposure and 10 (47.6%) were TKI-naïve, comparable to the distribution observed in the overall cohort (54.8% TKI-exposed).

### 3.2. Treatment Response

The overall ORR was 19.4% (95% CI, 9.2–36.3%; 6 partial responses), with a disease control rate (DCR) of 71.0% (95% CI, 53.4–83.9%) (Table 2). Response rates were consistent across treatment subgroups: ORR was similar between TKI-exposed (17.6%; 95% CI, 6.2–41.0%; *n* = 17) and TKI-naïve patients (21.4%; 95% CI, 7.6–47.6%; *n* = 14; *p* = 1.000), and between those receiving CEP as first-line (20.0%; 95% CI, 5.7–51.0%; *n* = 10) versus ≥2nd-line (19.0%; 95% CI, 7.7–40.0%; *n* = 21) therapy (*p* = 1.000; Table 2). A supplementary multivariable Cox model confirmed that line of CEP therapy was not independently associated with PFS (HR = 0.94; 95% CI, 0.40–2.26; *p* = 0.896) or OS (HR = 0.99; 95% CI, 0.37–2.60; *p* = 0.976; Supplementary Table S1). When stratified by ACC molecular subtype, objective responses occurred exclusively in ACC-I patients (6 of 17; ORR 35.3%, 95% CI 17.3–58.7%) compared with none in ACC-II (0 of 14; ORR 0%, 95% CI 0.0–21.5%; *p* = 0.021); DCR did not differ significantly between subtypes (76.5% vs. 64.3%; *p* = 0.693), and ACC-I patients received more treatment cycles (median 5 vs. 3). The subtype-specific response pattern was consistent across line of CEP therapy (ACC-I: 33.3% in first-line vs. 36.4% in ≥2nd-line; ACC-II: 0% in both), suggesting that treatment-line heterogeneity may not fully explain the observed subtype-associated response pattern.

**Figure 2:**
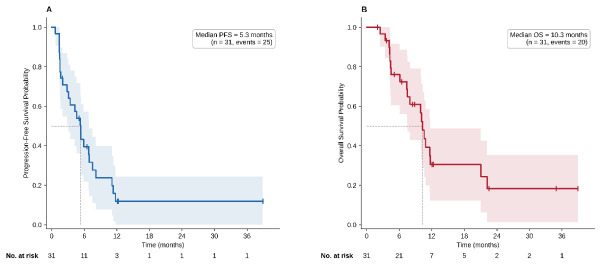
Kaplan–Meier estimates of progression-free survival (**A**) and overall survival (**B**) in the overall cohort (*n* = 31). Median PFS was 5.3 months; median OS was 10.3 months. Tick marks indicate censored observations; the number of patients at risk at each time point is shown below each plot.

**Figure 3:**
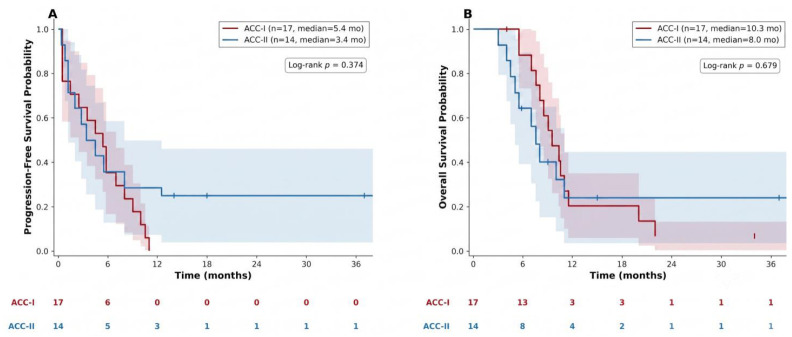
Kaplan–Meier estimates of progression-free survival (**A**) and overall survival (**B**) stratified by ACC molecular subtype in the overall cohort (*n* = 31). Median PFS was 5.4 months for ACC-I versus 3.4 months for ACC-II (log-rank *p* = 0.374); median OS was 10.3 versus 8.0 months (log-rank *p* = 0.679). Tick marks indicate censored observations; the number of patients at risk at each time point is shown below each plot. ACC, adenoid cystic carcinoma.

With a median follow-up of 22.6 months, median PFS and OS for the entire cohort were 5.3 and 10.3 months, respectively (Table 2, Figure 2A,B). No significant difference in PFS or OS was observed between ACC molecular subtypes (median PFS: ACC-I 5.4 vs. ACC-II 3.4 months, *p* = 0.374; median OS: 10.3 vs. 8.0 months, *p* = 0.679) (Table 2, Figure 3A,B).

### 3.3. Prognostic Factor Analysis in the Overall Cohort

Univariable Cox regression for PFS and OS was performed across nine clinical variables in the overall cohort (*n* = 31). No variable reached statistical significance for either endpoint (all *p* > 0.05; Supplementary Table S2). Multivariable analysis using Firth’s penalized likelihood Cox regression with pre-specified covariates (liver metastasis, bone metastasis, and prior TKI exposure) confirmed that prior TKI exposure was not independently associated with PFS (HR = 0.76; 95% CI, 0.33–1.74; *p* = 0.519) or OS (HR = 0.86; 95% CI, 0.33–2.23; *p* = 0.760). Bone metastasis showed a trend toward shorter PFS (HR = 2.43; 95% CI, 0.92–6.41; *p* = 0.074) but did not reach significance for OS (HR = 2.04; 95% CI, 0.63–6.55; *p* = 0.233) (Supplementary Table S3, Supplementary Figure S1A,B).

### 3.4. Prognostic Factor Analysis in the NGS Subgroup

**Figure 4:**
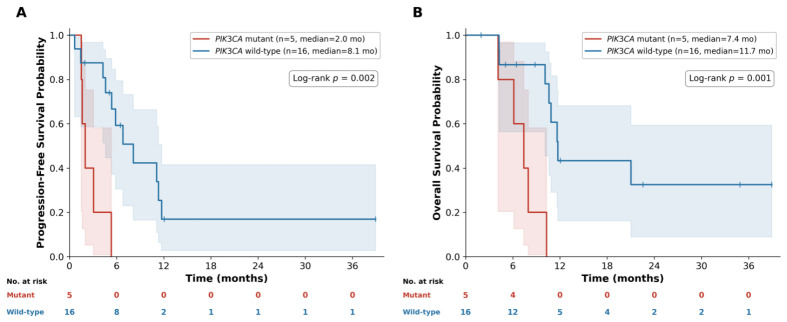
Kaplan–Meier estimates of progression-free survival (**A**) and overall survival (**B**) stratified by PIK3CA mutation status in the NGS subgroup (*n* = 21). PIK3CA-mutant patients (*n* = 5) versus PIK3CA-wild-type patients (*n* = 16). Median PFS was 2.0 versus 8.1 months (log-rank *p* = 0.006); median OS was 7.4 versus 11.7 months (log-rank *p* = 0.006). Tick marks indicate censored observations; the number of patients at risk at each time point is shown below each plot.

In the NGS subgroup (*n* = 21; 16 PFS events, 13 OS events), univariable Cox regression across clinical variables and the three most frequent genomic alterations identified PIK3CA mutation (present in 5 of 21 patients) and bone metastasis as potential prognostic signals requiring validation. PIK3CA mutation was associated with substantially shorter PFS (median 2.0 vs. 8.1 months; HR = 6.89; 95% CI, 1.75–27.13; *p* = 0.006) and OS (median 7.4 vs. 11.7 months; HR = 7.89; 95% CI, 1.82–34.23; *p* = 0.006) (Figure 4A,B). Bone metastasis was also associated with reduced PFS (median 2.0 vs. 6.8 months; HR = 3.50; 95% CI, 1.14–10.77; *p* = 0.029) and OS (median 10.1 vs. 11.7 months; HR = 3.70; 95% CI, 1.01–13.58; *p* = 0.049) (Supplementary Figure S2A,B). No other clinical or molecular variable reached significance (Table 3).

On multivariable Firth penalized Cox regression in this 21-patient subgroup, with CEP line (≥2nd-line vs. 1st-line), PIK3CA mutation, and bone metastasis included as covariates, PIK3CA mutation and bone metastasis remained associated with outcomes after adjustment, while CEP line was not. PIK3CA mutation was associated with shorter PFS (HR = 6.10; 95% CI, 1.29–28.81; *p* = 0.023) and OS (HR = 6.19; 95% CI, 1.27–30.14; *p* = 0.024). Bone metastasis was associated with shorter PFS (HR = 3.99; 95% CI, 1.10–14.50; *p* = 0.035) and OS (HR = 5.84; 95% CI, 1.22–28.02; *p* = 0.027). CEP line was not independently associated with PFS (HR = 0.62; 95% CI, 0.15–2.49; *p* = 0.499) or OS (HR = 0.36; 95% CI, 0.08–1.58; *p* = 0.175) (Table 4).

### 3.5. Safety

The median number of treatment cycles administered was 4 (range, 1–6). Prophylactic granulocyte colony-stimulating factor (G-CSF) was given to 25 patients (80.6%). The most common grade 3–4 hematologic toxicities were neutropenia (25.8%), anemia (19.4%), and thrombocytopenia (9.7%). Grade 3–4 nausea and vomiting occurred in 9.7% and 6.5% of patients, respectively. No adverse event led to treatment discontinuation, and no treatment-related deaths were observed.

## 4. Discussion

In this retrospective study of 31 patients with progressive R/M ACC treated with the CEP regimen, we observed an ORR of 19.4%, a disease control rate of 71.0%, a median PFS of 5.3 months, and a median OS of 10.3 months. The safety profile was manageable, and no treatment-related deaths occurred. These efficacy outcomes fall within the expected range for platinum-based combination chemotherapy in ACC. A systematic review of 25 chemotherapy trials reported single-agent ORRs of approximately 12%, while the CAP regimen achieved ORRs of approximately 25% across multiple studies [10]. In the largest of these, Licitra et al. reported partial responses in 27% of 22 patients with a median OS of 34 months [11]; that cohort, however, was chemotherapy-naïve and included patients without documented progression. The MD Anderson analysis—the largest real-world chemotherapy dataset in ACC (48 patients evaluable for response per RECIST)—reported an ORR of 12.5% and a median PFS of 4.3 months [20], further confirming these modest response rates. Such variability across studies likely reflects differences in patient selection, disease burden, prior treatment history, and progression criteria, precluding direct cross-study comparison.

Prior TKI exposure was not associated with reduced CEP efficacy: ORRs were 17.6% in TKI-pretreated versus 21.4% in TKI-naïve patients (*p* = 1.000), and multivariable analysis confirmed no independent association between prior TKI use and PFS or OS. This finding is concordant with the MD Anderson data showing comparable survival between first-line and later-line chemotherapy recipients [20], and supports platinum-based chemotherapy as a rational salvage option following TKI failure. Other cisplatin-containing regimens have reported activity in ACC, including cisplatin plus vinorelbine (ORR 32%) [21] and cisplatin plus docetaxel (ORR 23% in ACC) [22]; no head-to-head trial has compared any two regimens in R/M ACC, and the relative merits of these approaches remain undefined. Several caveats nonetheless apply: this comparison is non-randomized, and patients selected for CEP after TKI failure may have differed biologically from TKI-naïve patients—a classic confounding-by-indication scenario; in addition, detailed prior-TKI parameters (duration, best response, reason for discontinuation, washout interval) were inconsistently documented across the cohort and could not be analyzed systematically (Section 2.2). A supplementary Cox model further showed that line of CEP therapy itself was not independently associated with survival (Supplementary Table S1), suggesting that line of therapy alone may not fully explain these findings.

When stratified by ACC molecular subtype, all six partial responses occurred exclusively among ACC-I patients (ORR 35.3% vs. 0% in ACC-II; *p* = 0.021)—a pattern that, if confirmed in prospective studies, could have implications for patient selection. Disease control rates did not differ between subtypes (76.5% vs. 64.3%; *p* = 0.693), with the subtype-related difference confined to objective response. Although the high proportion of unspecified histology (51.6%) in our cohort precluded a histology–molecular subtype correlation analysis, the higher response rate observed in ACC-I aligns directionally with the higher chemotherapy response of solid-type ACC reported in the MD Anderson cohort, given that solid histology is a morphologic feature of ACC-I [16]. The MD Anderson data, however, also revealed a clinically important paradox: solid-type ACC—though more likely to respond to chemotherapy (ORR 20% vs. 6.7%)—had significantly shorter PFS (4.0 vs. 7.3 months; *p* = 0.018) and OS (10.8 vs. 24.1 months; *p* = 0.015) than non-solid tumors [20]. This dissociation between response and survival, which we also observed (ACC-I ORR 35.3% but no PFS/OS advantage), cautions against equating higher objective response with superior clinical benefit in ACC. The small number of responders (*n* = 6) in our cohort therefore precludes firm conclusions; prospective validation in molecularly stratified trials remains essential before any subtype-directed treatment selection.

ACC-I tumors are characterized by c-MYC overexpression with absent p63—a subtype defined by NOTCH pathway activation and MYC-driven transcription [16,17]. Although quantitative MYC expression and MYC genomic status were not directly examined in our cohort, indirect evidence from other solid tumors supports a potential link between MYC status and chemosensitivity: MYC amplification or overexpression has been associated with response to anthracycline-, cyclophosphamide-, and platinum-based chemotherapy in breast and ovarian cancers [23,24,25], and preclinical data demonstrate enhanced cytotoxicity of topoisomerase inhibitors and DNA-damaging agents in MYC-overexpressing cells [26,27]. The CEP regimen combines an alkylating agent, a topoisomerase II inhibitor, and a platinum compound—drug classes that, in these external datasets, have shown enhanced activity in MYC-overexpressing contexts. Whether this mechanism contributes to the response pattern observed in ACC-I patients in our cohort remains a hypothesis to be tested, requiring prospective studies with paired MYC expression and response data in larger ACC populations.

ACC-I nonetheless carries an inherently worse prognosis. In a molecular profiling study of 438 ACC tumors, ACC-I patients had significantly inferior overall survival compared with ACC-II, regardless of treatment modality [17]. A similar disparity was seen in a prospective phase II regorafenib trial (38 patients), where activating NOTCH1 or KDM6A alterations—markers of aggressive biology, NOTCH1 being a genomic hallmark of ACC-I—were associated with significantly shorter PFS (HR 2.6; *p* = 0.03), while clinical benefit was instead associated with immune-related gene signatures [28]. A differential pattern has also emerged in TKI-based trials: in the axitinib–avelumab phase II trial, ACC-II tumors showed a numerically higher disease control rate and more favorable PFS than ACC-I [29]. Mechanistically [16], ACC-II tumors show upregulation of TP63 with heterogeneous activation of receptor tyrosine kinases (AXL, EGFR, MET), making them dependent on RTK-driven signaling directly targeted by VEGFR-TKIs, whereas ACC-I tumors are driven primarily by MYC transcriptional amplification downstream of NOTCH activation—a pathway largely orthogonal to VEGFR signaling [16,30]. These mechanistic differences raise the hypothesis that ACC molecular subtype might eventually help inform, but cannot currently guide, the choice between cytotoxic chemotherapy and VEGFR-TKI therapy. This possibility requires direct testing in prospective comparative trials and should not influence current treatment decisions.

In the NGS subgroup (*n* = 21), multivariable analysis—adjusted for CEP line of therapy, PIK3CA mutation status, and bone metastasis—identified bone metastasis and PIK3CA mutation as potential prognostic signals, while CEP line was not independently associated with outcome. The prognostic significance of metastatic site in ACC is well recognized; Sung et al. demonstrated that patients with bone involvement had significantly worse survival than those with lung-only metastases [31], and a European multicenter study confirmed metastatic site as an independent variable in a validated nomogram [32]. This association may relate to the genomic profile of aggressive ACC—Ferrarotto et al. reported that activating NOTCH1 mutations define a subset characterized by solid histology, propensity for bone and liver metastasis, and dramatically worse prognosis [33]. In our NGS subgroup, a similar numerical trend was observed: bone metastasis was present in 50% (3/6) of NOTCH1-mutant patients versus 33% (5/15) of NOTCH1-wild-type patients, a direction consistent with prior reports but not reaching significance (*p* = 0.631) given the limited sample.

The PIK3CA mutation rate in our NGS subgroup was 23.8% (5/21), substantially higher than the 5% reported in the landmark whole-exome sequencing study of 60 ACC tumor-normal pairs [34]. Our cohort’s exclusive composition of patients with progressive R/M disease likely explains this difference; a subsequent genomic study demonstrated that recurrent and metastatic ACC tumors harbor higher mutational burden and greater frequency of PI3K pathway alterations compared with treatment-naïve primary tumors [35]. In this cohort, PIK3CA mutation was not associated with ACC molecular subtype in our data (ACC-I 15.4% vs. ACC-II 37.5%; *p* = 0.325), nor with NOTCH1 mutation status (*p* = 1.000), suggesting that it may be distributed independently of the NOTCH–MYC axis. Broader evidence implicating the PI3K/AKT pathway in ACC has been reported: Ouyang et al. reported that low p-mTOR expression was independently associated with locoregional recurrence and inferior survival in 120 resected ACC [36], and abnormal Akt activation has been correlated with aggressive biological behavior including enhanced perineural invasion [37]. Preclinically, a PI3K inhibitor effectively suppressed tumor growth in models harboring PIK3CA mutations [38]; in a small ACC subset enrolled in PI3K basket trials, 5 of 6 PIK3CA-mutant patients achieved disease control, with one partial response [35]. Whether this strategy can be applied in ACC requires dedicated prospective trials.

This study has several important limitations. The retrospective, single-center design and small sample size (*n* = 31) limit statistical power and generalizability. In particular, the NGS-subgroup multivariable Cox model was fitted with only 13 OS events across 3 covariates (events-per-variable = 4.3), below the conventional threshold of 10; NGS subgroup analyses are therefore subject to both limited statistical power and potential ascertainment bias (data available for 21 of 31 patients, 67.7%), and these estimates should be regarded as exploratory. The PIK3CA-associated outcome estimates are based on only 5 mutated patients with wide confidence intervals that span an order of magnitude and should therefore be interpreted with extreme caution and considered hypothesis-generating only. Molecular subtype assignments were reconstructed from c-MYC/p63 IHC results reported by referring institutions, without central pathology re-review and with inconsistent IHC positivity thresholds (Section 2.3); this represents a potential source of misclassification bias. Histologic subtype was unavailable in 51.6% of cases, limiting interpretation of the relationship between histology, molecular subtype, and treatment response. The cohort was clinically heterogeneous, with CEP administered across different treatment lines and preceded by variable prior therapies; the limited availability of detailed prior-TKI parameters (further discussed in the Section 4) likewise constrains TKI-related conclusions. Recurrence/metastatic TNM staging was not entered as a covariate in the survival models owing to uniform M1 status (93.5%) and inconsistent T/N retrieval from outside-institution records. Finally, response assessment was investigator-based rather than centrally reviewed, and no direct prospective comparison between CEP and VEGFR-targeted TKIs was performed; the subtype-directed treatment model proposed here therefore remains hypothesis-generating.

## 5. Conclusions

In this retrospective single-center study, CEP demonstrated activity (ORR 19.4%) with a manageable safety profile in progressive R/M ACC, and prior TKI exposure was not associated with reduced CEP efficacy in this cohort. Exploratory analyses suggest higher chemotherapy response rates in ACC-I patients and identify PIK3CA mutation as a potential adverse prognostic signal; these hypothesis-generating findings are not clinically actionable, and ACC molecular subtype or PIK3CA status should not be used for treatment selection based on these data alone. Prospective, molecularly stratified studies are required before these findings can inform clinical decision-making.

## Figures and Tables

**Table 1:** Baseline characteristics of patients (*n* = 31).

Variables	All Patients (*n* = 31)
Age, median, years (range)	48 (25–76)
Sex, *n* (%)	
Male	15 (48.4)
Female	16 (51.6)
ECOG PS, *n* (%)	
0	8 (25.8)
1	23 (74.2)
Primary site, *n* (%)	
Minor salivary gland	17 (54.8)
Major salivary gland	12 (38.7)
Lacrimal gland	2 (6.5)
Histology, *n* (%)	
Solid component	10 (32.3)
Cribriform/tubular	5 (16.1)
Unknown	16 (51.6)
Molecular subtypes, *n* (%)	
ACC-I	17 (54.8)
ACC-II	14 (45.2)
Disease distribution, *n* (%)	
Locoregional disease only	2 (6.5)
Metastatic disease only	13 (41.9)
Locoregional and metastatic disease	16 (51.6)
Initial presentation, *n* (%)	
De novo metastatic (Stage IV at diagnosis)	4 (12.9)
Recurrence after initial curative-intent treatment	27 (87.1)
Primary treatment, *n* (%)	
Surgery + adjuvant radiotherapy	22 (71.0)
Surgery alone	1 (3.2)
Definitive radiotherapy alone	4 (12.9)
No primary curative-intent treatment	4 (12.9)
Metastasis sites, *n* (%)	
Lung	20 (64.5)
Bone	10 (32.3)
Liver	9 (29.0)
Others	6 (19.4)
Prior lines of systemic treatment, *n* (%)	
0	10 (32.3)
1	14 (45.1)
≥2	7 (22.6)
Prior VEGFR TKI	17 (54.8)
Specific prior VEGFR TKI agents, *n* (%) *	
Anlotinib	9 (29.0)
Apatinib	8 (25.8)
Lenvatinib	3 (9.7)
Other prior systemic therapy classes, *n* (%) *	
Cytotoxic chemotherapy	7 (22.6)
Anti-PD-1 monoclonal antibody	4 (12.9)
Anti-EGFR monoclonal antibody	2 (6.5)
Investigational/clinical trial drug	2 (6.5)

Values are presented as *n* (%) unless otherwise indicated. * Frequencies in these categories may sum to more than the total number of patients with prior systemic therapy because some patients received drugs from multiple classes or were exposed to multiple TKIs across lines of therapy. ACC, adenoid cystic carcinoma; ECOG PS, Eastern Cooperative Oncology Group performance status; TKI, tyrosine kinase inhibitor; VEGFR, vascular endothelial growth factor receptor.

**Table 2:** Treatment Response and Survival Outcomes by ACC Molecular Subtype, Prior TKI Exposure, and Line of CEP Therapy in the overall cohort (*n* = 31).

Variables	All Patients (*n* = 31)	ACC-I (*n* = 17)	ACC-II (*n* = 14)	*p* Value	Prior TKI− (*n* = 14)	Prior TKI+ (*n* = 17)	*p* Value	CEP as 1st-Line (*n* = 10)	CEP as ≥2nd-Line (*n* = 21)	*p* Value
Cycles, median (range)	4 (1–6)	5 (1–6)	3 (2–6)	—	4 (2–6)	4 (1–6)	—	3.5 (2–6)	4 (1–6)	—
Best overall response, *n* (%)				—			—			—
PR	6 (19.4)	6 (35.3)	0 (0)		3 (21.4)	3 (17.6)		2 (20.0)	4 (19.0)	
SD	16 (51.6)	7 (41.2)	9 (64.3)		6 (42.9)	10 (58.8)		4 (40.0)	12 (57.1)	
PD	9 (29.0)	4 (23.5)	5 (35.7)		5 (35.7)	4 (23.5)		4 (40.0)	5 (23.8)	
ORR (%) (95%CI)	19.4 (9.2–36.3)	35.3 (17.3–58.7)	0.0 (0.0–21.5)	0.021	21.4 (7.6–47.6)	17.6 (6.2–41.0)	1.000	20.0 (5.7–51.0)	19.0 (7.7–40.0)	1.000
DCR (%) (95%CI)	71.0 (53.4–83.9)	76.5 (52.7–90.4)	64.3 (38.8–83.7)	0.693	64.3 (38.8–83.7)	76.5 (52.7–90.4)	0.693	60.0 (31.3–83.2)	76.2 (54.9–89.4)	0.417
Median follow-up, months	22.6	34.9	12.3		34.9	22.6	—	12.3	22.6	—
Median PFS, months	5.3	5.4	3.4	0.374	5.3	5.4	0.877	5.3	5.4	0.906
Median OS, months	10.3	10.3	8.0	0.679	11.6	10.1	0.888	10.3	10.1	0.963

ACC-I (*n* = 17), ACC-II (*n* = 14); CEP as first-line (*n* = 10), CEP as ≥2nd-line (*n* = 21). CEP, cyclophosphamide, epirubicin, and cisplatin; CI, confidence interval; DCR, disease control rate; OS, overall survival; ORR, objective response rate; PD, progressive disease; PFS, progression-free survival; PR, partial response; SD, stable disease.

**Table 3:** Univariable Cox Proportional Hazards Regression for PFS and OS in the NGS Subgroup (*n* = 21).

	Progression-Free Survival					Overall Survival				
Variable	Median (+)	Median (−)	HR	95% CI	*p* Value	Median (+)	Median (−)	HR	95% CI	*p* Value
ECOG PS (1 vs. 0)	5.3	6.8	1.954	0.529–7.220	0.315	10.3	11.7	2.486	0.534–11.565	0.246
Sex (female vs. male)	5.4	5.9	1.041	0.376–2.884	0.938	10.9	10.6	0.646	0.216–1.935	0.435
Primary site (minor vs. major SG)	5.4	4.6	0.762	0.284–2.045	0.589	10.3	10.9	0.803	0.268–2.410	0.696
Solid component (yes vs. no)	5.3	5.9	1.478	0.532–4.104	0.453	10.3	10.9	1.080	0.348–3.356	0.894
Lung metastasis (yes vs. no)	5.9	2.0	0.528	0.190–1.468	0.221	11.6	10.1	0.605	0.191–1.916	0.393
Liver metastasis (yes vs. no)	8.1	5.4	1.002	0.339–2.957	0.998	10.6	11.6	1.053	0.319–3.476	0.933
Bone metastasis (yes vs. no)	2.0	6.8	3.502	1.138–10.774	0.029	10.1	11.7	3.696	1.006–13.576	0.049
Prior TKI (yes vs. no)	6.8	5.3	0.573	0.206–1.599	0.288	10.9	10.3	0.564	0.188–1.685	0.305
CEP line (≥2nd-line vs. 1st-line)	5.9	5.3	0.711	0.221–2.283	0.566	10.9	10.3	0.476	0.145–1.560	0.220
ACC subtype (I vs. II)	5.4	6.8	2.376	0.735–7.673	0.148	10.6	11.7	1.843	0.557–6.095	0.317
NOTCH1 mutation (yes vs. no)	5.3	6.8	1.415	0.453–4.421	0.551	10.3	11.7	2.550	0.724–8.984	0.145
PIK3CA mutation (yes vs. no)	2.0	8.1	6.886	1.748–27.130	0.006	7.4	11.7	7.891	1.819–34.227	0.006
MYB fusion (yes vs. no)	5.9	3.1	0.743	0.265–2.084	0.572	10.9	8.0	0.953	0.289–3.136	0.937

Median (+) and Median (−) denote median survival (months) with and without the indicated variable, respectively. ACC, adenoid cystic carcinoma; CI, confidence interval; ECOG PS, Eastern Cooperative Oncology Group performance status; HR, hazard ratio; SG, salivary gland; TKI, tyrosine kinase inhibitor.

**Table 4:** Multivariable Firth Penalized Cox Regression for PFS and OS in the NGS Subgroup (*n* = 21).

	Progression-Free Survival				Overall Survival			
Variable	HR	SE	95% CI	*p* Value	HR	SE	95% CI	*p* Value
CEP line (≥2nd-line vs. 1st-line)	0.619	0.7092	0.154–2.485	0.499	0.357	0.7583	0.081–1.580	0.175
PIK3CA mutation (yes vs. no)	6.095	0.7925	1.290–28.808	0.023	6.188	0.8078	1.271–30.138	0.024
Bone metastasis (yes vs. no)	3.993	0.6579	1.100–14.498	0.035	5.844	0.7998	1.219–28.019	0.027

Events: 16/21 (76.2%) for progression-free survival and 13/21 (61.9%) for overall survival. Events-per-variable were 5.3 for PFS and 4.3 for OS. Analyses were performed using Firth’s penalized likelihood Cox regression to reduce finite-sample bias. Estimates are exploratory given the limited sample size. Pre-specified covariates were CEP line (≥2nd-line vs. 1st-line), PIK3CA mutation status, and presence of bone metastasis. CI, confidence interval; HR, hazard ratio; OS, overall survival; PFS, progression-free survival; SE, standard error.

## Data Availability

The raw data supporting the conclusions of this article will be made available by the authors on request.

## Supplementary Materials

The following supporting information can be downloaded at: https://www.mdpi.com/article/10.3390/cancers18111847/s1, Table S1: Multivariable Cox Regression—ACC Subtype + Treatment Line (*n* = 31); Table S2: Univariable Cox Proportional Hazards Regression for PFS and OS (*n* = 31); Table S3: Multivariable Firth Penalized Cox Regression for PFS and OS (*n* = 31); Figure S1: Kaplan–Meier estimates of progression-free survival (A) and overall survival (B) stratified by prior TKI exposure in the overall cohort (*n* = 31). TKI-exposed patients (*n* = 17) versus TKI-naïve patients (*n* = 14). Median PFS was 5.4 months (TKI-exposed) versus 5.3 months (TKI-naïve; log-rank *p* = 0.877); median OS was 10.1 versus 11.6 months (log-rank *p* = 0.888). Tick marks indicate censored observations; the number of patients at risk at each time point is shown below each plot. TKI, tyrosine kinase inhibitor; Figure S2: Kaplan–Meier estimates of progression-free survival (A) and overall survival (B) stratified by bone metastasis status in the NGS subgroup (*n* = 21). Patients with bone metastasis (*n* = 6) versus those without (*n* = 15). Median PFS was 2.0 versus 6.8 months (log-rank *p* = 0.029); median OS was 10.1 versus 11.7 months (log-rank *p* = 0.049). Tick marks indicate censored observations; the number of patients at risk at each time point is shown below each plot.
