# Supplementary material for: Molecular Subtype-Associated Response to Cyclophosphamide–Epirubicin–Cisplatin Regimen in Recurrent or Metastatic Adenoid Cystic Carcinoma: A Retrospective Single-Center Study
Source: Cancers (Basel). 2026 Jun 4;18(11):1847. doi: 10.3390/cancers18111847 (PMC13256737; doi:10.3390/cancers18111847)
Supplement: Supplementary file 1 [file cancers-18-01847-s001.zip › cancers-4340571-supplementary.pdf]

Supplementary Tables and Figures

Supplementary Table S1. Multivariable Cox Regression — ACC Subtype + Treatment Line (N=31)

	PFS				OS			
**Variable**	**HR**	**SE**	**95% CI**	***P* value**	**HR**	**SE**	**95% CI**	***P* value**
ACC subtype (I vs II)	1.437	0.426	0.623–3.319	0.395	1.208	0.464	0.487–3.000	0.683
Treatment line (1st-line vs ⩾2nd-line)	0.944	0.445	0.395–2.257	0.896	0.985	0.495	0.374–2.597	0.976

Events: 25/31 (80.6%) for progression-free survival and 20/31 (64.5%) for overall survival. Events-per-variable were 12.5 for PFS and 10.0 for OS. Analyses were performed using Firth's penalized likelihood Cox regression, consistent with other multivariable models in the main analysis. Estimates are exploratory given the limited sample size. "CEP as 1st-line" refers to patients receiving CEP as initial systemic therapy without prior systemic treatment; "CEP as ⩾2nd-line" refers to patients who had received one or more prior systemic regimens. ACC, adenoid cystic carcinoma; CEP, cyclophosphamide, epirubicin, and cisplatin; CI, confidence interval; HR, hazard ratio; OS, overall survival; PFS, progression-free survival.

Supplementary Table S2. Univariable Cox Proportional Hazards Regression for PFS and OS (N=31)

	**Progression-Free Survival**					**Overall Survival**				
Variable	Median (+)	Median (−)	HR	95% CI	*P* Value	Median (+)	Median (−)	HR	95% CI	*P* Value
ECOG PS (1 vs 0)	5.3	3.4	1.579	0.609–4.095	0.347	10.1	11.7	1.762	0.577–5.381	0.320
Sex (female vs. male)	5.3	4.3	0.948	0.431–2.086	0.894	10.9	8.0	0.462	0.184–1.158	0.100
Primary site (minor vs. major SG)	5.4	4.6	0.758	0.344–1.666	0.490	10.1	10.9	0.950	0.394–2.294	0.910
Solid component (yes vs. no)	5.4	4.6	1.172	0.517–2.657	0.705	9.9	10.9	1.352	0.545–3.351	0.515
Lung metastasis (yes vs. no)	5.9	2.0	0.513	0.226–1.161	0.109	11.6	7.4	0.478	0.191–1.195	0.114
Liver metastasis (yes vs. no)	7.5	5.3	0.872	0.359–2.115	0.761	8.0	10.3	1.215	0.461–3.200	0.693
Bone metastasis (yes vs. no)	4.6	5.9	1.793	0.759–4.236	0.183	10.1	11.6	1.870	0.679–5.150	0.226
Prior TKI (yes vs. no)	5.4	5.3	0.939	0.424–2.082	0.877	10.1	11.6	1.066	0.440–2.582	0.888
CEP line (≥2nd-line vs. 1st-line)	5.4	5.3	1.056	0.439–2.539	0.904	10.1	10.3	0.977	0.374–2.553	0.963
ACC subtype (I vs. II)	5.4	3.4	1.459	0.628–3.391	0.380	10.3	8.0	1.209	0.491–2.978	0.680

Median (+) and Median (−) denote median survival (months) with and without the indicated variable, respectively. ACC, adenoid cystic carcinoma; CI, confidence interval; ECOG PS, Eastern Cooperative Oncology Group performance status; HR, hazard ratio; SG, salivary gland; TKI, tyrosine kinase inhibitor.

Supplementary Table S3. Multivariable Firth Penalized Cox Regression for PFS and OS (N=31)

	Progression-Free Survival				Overall Survival			
**Variable**	**HR**	**SE**	**95% CI**	***P* value**	**HR**	**SE**	**95% CI**	***P* value**
Liver metastasis (yes vs. no)	0.636	0.4940	0.242–1.675	0.360	0.968	0.5353	0.339–2.764	0.952
Bone metastasis (yes vs. no)	2.425	0.4957	0.918–6.408	0.074	2.037	0.5960	0.633–6.551	0.233
Prior TKI (yes vs. no)	0.761	0.4227	0.333–1.744	0.519	0.863	0.4836	0.334–2.226	0.760

Events: 25/31 (80.6%) for progression-free survival and 20/31 (64.5%) for overall survival. Events-per-variable were 8.3 for PFS and 6.7 for OS. Analyses were performed using Firth's penalized likelihood Cox regression to reduce finite-sample bias. Estimates are exploratory given the limited sample size. Pre-specified covariates were liver metastasis, bone metastasis, and prior TKI exposure. CEP line of therapy is strongly correlated with prior TKI exposure in this cohort (all 17 prior-TKI patients received CEP at ≥2nd line; all 10 1st-line CEP patients are TKI-naïve) and was therefore evaluated in a separate multivariable model alongside ACC subtype (Supplementary Table S3) rather than being included in the present model. CI, confidence interval; HR, hazard ratio; OS, overall survival; PFS, progression-free survival; SE, standard error; TKI, tyrosine kinase inhibitor.

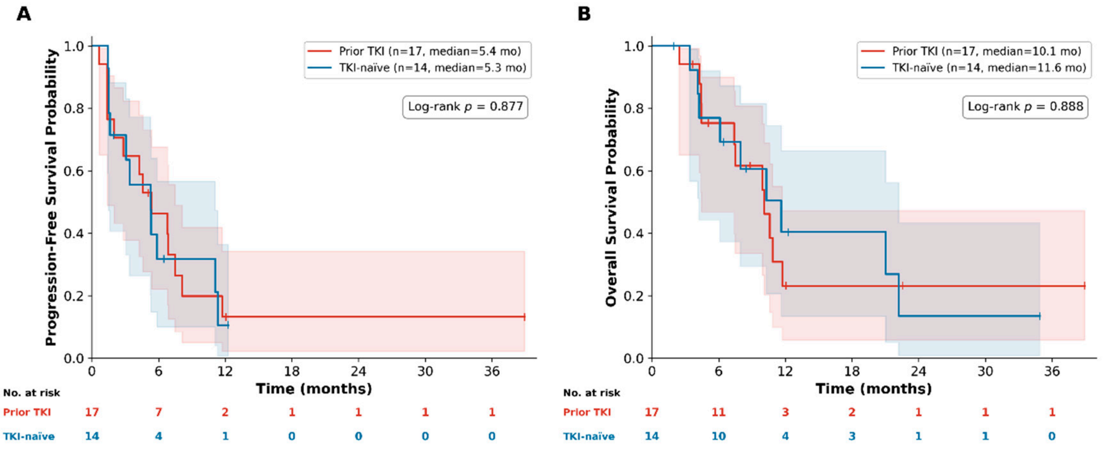

Supplementary Figure S1. Kaplan–Meier estimates of progression-free survival (A) and overall survival (B) stratified by prior TKI exposure in the overall cohort (N = 31). TKI-exposed patients (n = 17) versus TKI-naïve patients (n = 14). Median PFS was 5.4 months (TKI-exposed) versus 5.3 months (TKI-naïve; log-rank $P = 0.877$); median OS was 10.1 versus 11.6 months (log-rank $P = 0.888$). Tick marks indicate censored observations; the number of patients at risk at each time point is shown below each plot. TKI, tyrosine kinase inhibitor.

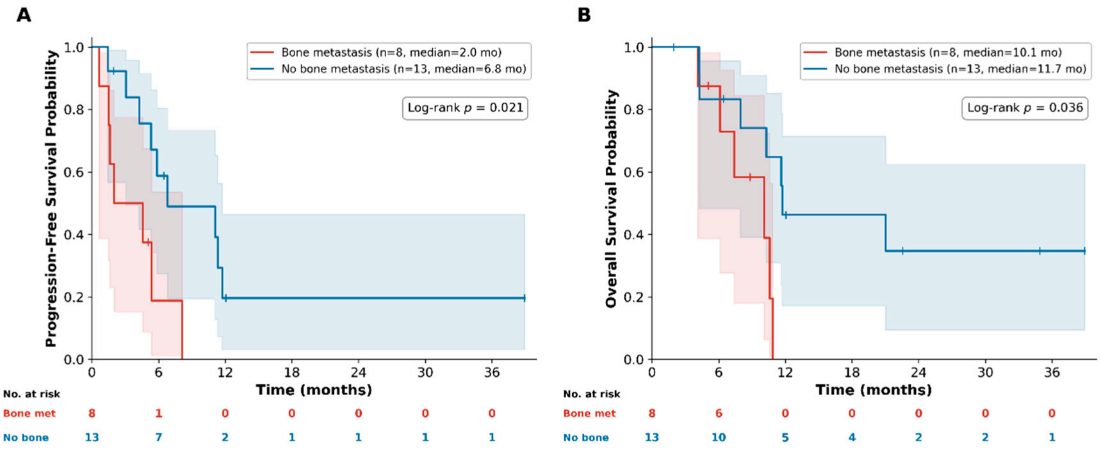

Supplementary Figure S2. Kaplan–Meier estimates of progression-free survival (A) and overall survival (B) stratified by bone metastasis status in the NGS subgroup (N = 21). Patients with bone metastasis (n = 6) versus those without (n = 15). Median PFS was 2.0 versus 6.8 months (log-rank $P = 0.029$); median OS was 10.1 versus 11.7 months (log-rank $P = 0.049$). Tick marks indicate censored observations; the number of patients at risk at each time point is shown below each plot.
